# Towards healthy settings for people with intellectual disabilities

**DOI:** 10.1093/heapro/daz054

**Published:** 2019-06-26

**Authors:** Kristel Vlot-van Anrooij, J Naaldenberg, T I M Hilgenkamp, L Vaandrager, K van der Velden, G L Leusink

**Affiliations:** 1 Department of Primary and Community Care, Research group Intellectual Disabilities and Health, Radboud Institute for Health Sciences, Radboud University Medical Center, PO Box 9101, 6500 HB Nijmegen, The Netherlands; 2 Department of General Practice, Intellectual Disability Medicine, Erasmus MC, University Medical Center Rotterdam, PO Box 2040, 3000CA Rotterdam, The Netherlands; 3 Department of Social Sciences, Health and Society, Wageningen University, PO Box 8130, 6706 KN Wageningen, The Netherlands; 4 Department of Primary and Community Care, Radboud Institute for Health Sciences, Radboud University Medical Center, PO Box 9101, 6500 HB Nijmegen, The Netherlands

**Keywords:** health promotion, healthy settings, intellectual disability, settings approach, concept mapping

## Abstract

People with intellectual disabilities (ID) depend on their environment for support to live healthily. The characteristics of healthy settings for people with ID are unknown. This study aims to conceptualize healthy settings for people with ID by conducting an international and multidisciplinary concept mapping study. As theoretical framework the settings approach, an ecological model with a whole system focus toward health promotion, was used. The integrative mixed-methods approach of this study involved concept mapping with researchers specialized in healthcare for people with ID and researchers specialized in healthy settings. The 41 participants generated statements that were later sorted and rated. Findings encompass 13 clusters relating to the social environment, the physical environment and societal preconditions. Specific factors of healthy settings for people with ID include: (i) universal design of the physical environment, (ii) the role of care professionals in the social environment to empower people with ID, (iii) possibilities for care providers to contribute to a health-promoting setting and (iv) preconditions that allow people to engage in society. These factors can be used in strategies to apply the approach in practice and give directions to put in place policies on developing enabling environments and decreasing health inequities.

## INTRODUCTION

Developing healthy settings has the potential to develop a supportive context within the places in which people engage and to support individuals to live a healthy life. The settings approach adopts an ecological model, meaning that there are dynamic interrelations between personal and environmental factors that promote or damage health. Settings are also viewed as complex systems, and the settings approach takes a whole system focus aimed at embedding health within routines and the culture of the setting (Dooris, 2013). In line with this dynamic view, health can be promoted if inside agents are given the capacity to address behavioral and environmental factors within the setting (Whitelaw *et al.*, 2001). The settings approach has been applied in many contexts, of which the Healthy Cities, Healthy Universities and Healthy School projects are well-known examples resulting in transformed policies, organizational structures and community action to facilitate healthy living and participation (Mũkoma and Flisher, 2004; Dooris *et al.*, 2012; Schwab *et al.*, 2015). People with intellectual disabilities (ID) are characterized by limitations in adaptive behavior, communication and cognitive processes (APA, 2013). Applying the settings approach in care settings where people with ID live, work and engage is expected to be beneficial to the health and well-being of people with ID for three major reasons.

Firstly, people with ID experience health inequalities, and face problems with accessing healthcare, prevention and health promotion (Beange and Durvasula, 2001; Cooper *et al.*, 2004; Van Schrojenstein Lantman-de Valk and Walsh, 2009; Heslop *et al.*, 2014). Reducing health inequalities by developing a healthy settings approach for people with ID is in line with the United Nations (UN) sustainable development goals on reducing inequities and promoting health and well-being, the UN convention on the rights of people with disabilities, and the World Health Organization (WHO) goals on increasing health equity and developing enabling environments for people with disabilities (WHO, 2008, 2011; United Nations, 2015a,b). The settings approach has been mainly applied in on contexts where vulnerable populations often do not engage and elaborating on this approach for people with ID addresses this imbalance (Whitelaw *et al.*, 2001).

Secondly, the settings approach should be applied in setting where people with ID engage. Specialized care providers have a considerable influence on the everyday life and living environment of people with ID. These specialized care providers can provide housing, help with daily living tasks, organized daytime or work activities, and medical care for people with ID (Ras *et al.*, 2013). However, the organizational culture of these care providers and the education of support staff is mainly centered on treating health problems rather than on health promotion (O’Leary *et al.*, 2018).

Thirdly, existing health promotion faces difficulties and research identified the need for developing a supportive context for healthy living. Traditional lifestyle interventions for the general population often do not reach people with ID, because many of them do not have the required independence, money and literacy skills to participate (Messent *et al.*, 2000; Robertson *et al.*, 2000). Health promotion efforts in care settings for people with ID and through care provider services are focused on individual behavior change, group behavior change and interpersonal support. These efforts have often failed to produce sustained health benefits over time (Heller *et al.*, 2011; Naaldenberg *et al.*, 2013; Scott and Havercamp, 2016). People with ID themselves have expressed the need for a supportive setting, including support from the social environment and facilities in the physical environment that enable healthy choices (Kuijken *et al.*, 2016). Setting-related factors, including support from others, embedment of health promotion policies in organizations for people with ID, and facilities for physical activity and healthy eating are mentioned in the literature as facilitators of healthy living (Temple and Walkley, 2007; Caton *et al.*, 2012; Bergström *et al.*, 2014; Sundblom *et al.*, 2015; Kuijken *et al.*, 2016) which implies a need for developing supportive contexts for health living of people with ID. To develop healthy settings for people with ID, account must be taken of the characteristics of the population, their support needs, their living environment and the core business of the setting (Dooris, 2016).

Our study aims to conceptualize healthy settings for people with ID. To better tailor health promotion for people with ID a multidisciplinary approach was chosen making use of the knowledge base on healthy settings and needs of people with ID (Dooris, 2016; Poland *et al.*, 2011, Naaldenberg *et al.*, 2013). To study factors that are perceived to be important for developing a healthy setting for people with ID, this study takes an international perspective in which researchers with experience in academic research and practice (development and delivery of care) participate in a concept mapping study.

## MATERIALS

### Study design

In this study, an integrative mixed method approach was used in which both quantitative and qualitative data were collected and combined in the analysis (Cresswell, 2013). The concept mapping method was used because it is specifically developed to explore complex concepts and generate conceptual frameworks (Trochim and Kane, 2005; Kane and Trochim, 2007). The method consists of two data collection phases: (i) brainstorming guided by focus prompts and (ii) sorting and rating of statements resulting from phase 1. Including the experts both in generating topics as well as in structuring the topics into clusters was expected to lead to a conceptualization of healthy settings which is reflective of perspectives of diverse groups (Kane and Trochim, 2007).

### Procedures

Expert sampling was used to select researchers who were involved either in healthcare for people with ID or in healthy settings. Names of potential participants were acquired from: (i) the conference proceedings of the health conference of the International Association for the Scientific Study of Intellectual and Developmental Disabilities (IASSIDD) in June 2017; (ii) members of the European Training Consortium in Public Health and Health Promotion; (iii) the network of members of the research team; and (iv) key authors with expertise in the field of healthcare for people with ID or field of healthy settings. A list of 66 potential participants was agreed among the research team.

For the brainstorming phase, live and online brainstorming were combined. Live brainstorming facilitates group interaction and focuses on the task, and online brainstorming allows people from different countries to participate (Kane and Trochim, 2007). Potential participants who attended the IASSIDD 2017 health conference were personally invited to participate in live brainstorming during the conference. Other potential participants received an email invitation to the online brainstorming. Prior to the live and the online brainstorming, the study information was repeated and informed consent was obtained. The live brainstorming session was voice-recorded. The participants in the brainstorming phase were invited to participate in the next phase of sorting and rating. Additional participants were recruited to include more healthy settings researchers. The data were collected between 21 June 2017 and 31 October 2017, supported by Concept System Global MAX software.

### Participants

The response rate for the brainstorming phase was 62% (*n* = 41) and to the sorting and rating phase 65% (*n* = 32). In the brainstorming phase, 7 participants participated in live brainstorming and 34 in online brainstorming. Their field of expertise was either healthy settings (*n* = 11 in the brainstorming and *n* = 6 in the sorting phase) or healthcare for people with ID (*n* = 30 in the brainstorming and *n* = 26 in the sorting phase). The participants had on average 16 years of research experience and 15 years of experience as a practitioner (development and delivery of care). Participants were resident in the UK (*n* = 12), the USA (*n* = 6), the Netherlands (*n* = 6), Canada (*n* = 3), Australia (*n* = 2), Ireland (*n* = 2), Norway (*n* = 2), Spain (*n* = 2), Chili (*n* = 1), Finland (*n* = 1), Germany (*n* = 1), Iceland (*n* = 1), Italy (*n* = 1) and Saudi-Arabia (*n* = 1).

### Data collection and analysis

The phases, actions and results of data collection and analysis are described in Table 1. The brainstorming phase was guided by focus prompts that participants were asked to finish in as many different ways as possible. The focus prompts used were: ‘I am a person with an intellectual disability and my setting looks like ….’ and ‘I am a person with an intellectual disability and my setting is promoting health by….’ During the live brainstorming, the participants wrote their statements finishing the focus prompt sentences on post-its and expressed ideas within the group. For the online brainstorming, statements were entered in the online system. To stimulate participants’ thinking process, previous participants’ statements were visible. The brainstorm phase resulted in 445 statements. As required by the procedure (Kane and Trochim, 2007), the statements were synthesized until a set of maximum 100 statements was reached. These statements were used in the sorting and rating phase (Table 1).

**Table 1:** Phases, actions and results of data collection and analysis

Phase	Action	Result
Preparation phase 1	Develop and pilot focus prompts to obtain information on ‘health promoting characteristics of the setting’ with research team (K.V.A., J.N., T.I.M.H., L.V., K.V., G.L.L.)	2 focus prompts
Phase 1: brainstorm	Create statements related to the focus prompts: One live brainstorming with seven researchers Online brainstorming with 34 researchers	455 statements
Preparation phase 2	Data synthesis of statements using the following procedure: Split up statements containing >1 statement per sentence Remove identical statements Assign keywords to the statements and sort to bring overlapping statements together Combine overlapping statements Participant check on reduced set by two participants	Statements reduced to a set of 100 statements
Phase 2: sorting and rating	32 participants sort statements in categories and rate statements on a 5-point Likert scale	100 statements individually sorted and rated
Data analysis	(A) *Multidimensional scaling:* create a point map based on the sorting data to visualize the relationship and proximity of statements to one another	(A) Point map
	(B) *Hierarchical cluster analysis:* create a cluster map: Decide upper and lower limits of clusters (K.V., J.N.) Assess individually what cluster size retains most useful detail between clusters by looking at the bridging values and how the clusters merge together when moving from the upper limit to the lower limit of the cluster sizes (K.V.A., J.N., T.I.M.H., L.V., K.V., G.L.L.) Choose final cluster size and names (examining cluster statements and top-10 cluster names generated by participants) (K.V.A., J.N., T.I.M.H., L.V., K.V., G.L.L.) Calculate stress value and bridging values for the concept map Estimate sensitivity of the concept map using jackknife	(B) Final cluster map
	(C) *Analyze importance ratings:* Calculate mean rate for statements and clusters	(C) Rating of statements and clusters

In the online sorting and rating phase, the participants sorted each of the 100 statements into a category based on how similar in meaning or theme they were and named the categories according to their content. Next, the participants rated each statement, on a 5-point Likert scale, on its importance for healthy settings for people with ID. Participants were asked to complete questions on country of residence, expert group, years of academic experience, years of experience as a practitioner, and whether they wished to be mentioned in the acknowledgements of this article.

Data analysis was conducted using Concept Systems Global MAX software. The software created a similarity matrix indicating the number of people who placed a statement in the same pile by using the group’s sorting data. This was analyzed using nonmetric multidimensional scaling, and a point map was created, representing the distances and relations between statements. Hierarchical cluster analysis was used to divide the map into clusters. To determine each cluster size and name, the procedure recommended by Kane and Trochim was used, see Table 1 (Kane and Trochim, 2007). A stress value was calculated for the cluster map; this gives an indication of the goodness of fit of the map to the original similarity matrix, where a lower value represents a better overall fit. Bridging values, indicating how much a statement is anchored to those around it or bridges with statements further away, were calculated for all statements and clusters in the cluster map (Kane and Trochim, 2007). The importance ratings of the statements were analyzed by calculating the mean rate for each statement and for all clusters. To investigate sensitivity for sampling variation the jackknife resampling method was used. The original distribution of statements within clusters was compared with the 31 distributions resulting from systematically leaving out one participant from the sample (delete one jackknife). The amount of statements that were placed in another cluster was calculated.

## RESULTS

This section presents the results of the brainstorming and the sorting of statements, including the concept map with a description of each cluster, the sensitivity of the concept map and the importance ratings of statements and clusters.

### Brainstorming and sorting of statements

The 100 statements that resulted from the brainstorming phase are presented in Supplementary Appendix S1. The participants sorted the statements on average in 9 clusters, with a minimum of 5 and a maximum of 16 clusters. In Supplementary Appendix S2, the point map is displayed, a visual representation of the relationship of the 100 statements to one another based on the sorting data of all participants.

### Concept map

**Fig. 1:**
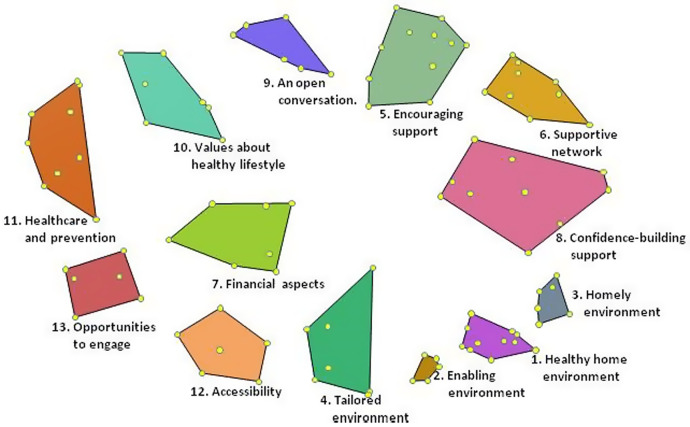
Final concept map: a spatial representation of how the 100 statements (dots) relate to the 13 clusters.

The final concept map includes 13 clusters. Figure 1 depicts how the statements relate in a spatial representation to these clusters. Items that are closer to one another are more closely related to one another. The cluster with the highest coherence contains 5 statements and the cluster with the lowest coherence contains 11 statements. The stress value of the final concept map was 0.32, which is similar to other concept mapping projects where stress values range between 0.21 and 0.37 (Kane and Trochim, 2007).

The 13 clusters have the following names: *Healthy home environment, Enabling environment, Homely environment, Tailored environment, Encouraging support, Supportive network, Financial aspects, Confidence-building support, An open conversation, Values about healthy lifestyle, Healthcare and prevention, Accessibility* and *Opportunities to engage*. Based on the statements within the clusters, for each cluster a definition was formulated by the research team (see Table 2).

**Table 2:** Clusters, descriptions, mean bridging values (B) and importance ratings (I)

Cluster (number of statements)	Description	B^a^	I^b^
1. Healthy home environment (11 statements)	A comfortable and attractive house with facilities for healthy living such as a kitchen, garden, room with daylight and nice views	0.07	3.79
2. Enabling environment (5 statements)	There are accessible places nearby that are inviting for physical activity and meeting people	0.14	4.17
3. Homely environment (6 statements)	A place you can call home, where you feel safe and can experience happiness	0.21	3.82
4. Tailored environment (7 statements)	The alignment and connectivity between an individual and his/her environment	0.35	3.33
5. Encouraging support (11 statements)	Support (tangible, emotional and companionship) from others that encourage a person to live a healthy life	0.36	4.05
6. Supportive network (9 statements)	Having people around you that can provide sufficient support	0.41	4.12
7. Financial aspects (7 statements)	Sufficient money for healthy food, healthy activities, adaptations and resources	0.45	3.86
8. Confidence-building support (10 statements)	A person gets personal space to enable independence and also receives the right amount of support and cues in daily life	0.49	4.11
9. An open conversation (6 statements)	A discussion about health topics in which everyone’s ideas are taken seriously	0.50	4.14
10. Values about healthy lifestyle (7 statements)	How other people think about healthy living for people with ID	0.56	4.08
11. Healthcare and prevention (9 statements)	Having access to health professionals providing person-centered medical care, health-related guidelines and attention to prevention	0.65	4.00
12. Accessibility (6 statements)	Visible and invisible things that make it possible to go to healthy activities, such as safety and absence of obstacles	0.65	3.78
13. Opportunities to engage (6 statements)	(Un)equal rights, control, power to influence, access, and (financial) dependence	0.91	3.66

a B = mean bridging value for clusters between 0 and 1.

b I = importance (rated on a 5-point Likert scale).

The majority of clusters has mean bridging values ranging between 0.35 and 0.65, representing an overall moderate level of cluster anchoring (Supplementary Appendix S1). Clusters *Healthy home environment* (0.07), *Enabling environment* (0.14) and *Homely environment* (0.21) have very low mean bridging values, indicating that the statements within these clusters are conceptually closely related to one another. The cluster *Opportunities to engage* (0.91) has a relatively high mean bridging value, which is a product of statements that were frequently grouped with items other than those in their immediate vicinity. Table 2 presents the clusters sorted by mean bridging value.

### Interpretation of concept map

The 13 identified clusters describe how the physical environment, the social environment and preconditions for healthy living in society can support health. Resources in the physical environment are described in the clusters *Healthy home environment* and *Enabling environment*. The clusters *Tailored environment* and *Accessibility* describe barriers and resources specifically for people with ID, which demonstrated the need for a fit between resources and needs of people with ID. The interconnectivity between the physical and social environment is visible in the cluster *Homely environment*, where statements related to places and people are included. The clusters relating to the social environment describe the social network (*Supportive network*) and prerequisites for it to be promoting health (*Values about healthy lifestyle, An open conversation*, *Confidence-building support* and *Encouraging support*). Notably is the role of the social network of people with ID to empower them. Preconditions for healthy living in society are described in three clusters (*Financial aspects*, *Healthcare and prevention* and *Opportunities to engage*) including access to healthy food and health professionals as well as (not) having the same opportunities as everyone else in society*.* Besides, several opportunities for care providers to contribute to healthy settings were mentioned in the 13 clusters.

### Importance of statements and clusters

Table 2 presents the importance of the statements at cluster level; the importance of each statement is indicated in Supplementary Appendix S1. The importance of the statements ranged from 2.72 to 4.78. The importance ratings of the clusters were denser and ranged from 3.33 (*Tailored environment*) to 4.17 (*Enabling environment*).

### Sensitivity of concept map

To investigate the sensitivity of the concept map to sampling variation, the jackknife resampling method was applied. Comparison of the jackknife distributions (of statements within clusters) and the original distribution revealed that, on average, 17 of the 100 statements were placed in another cluster than in the original distribution. At cluster level, some of the jackknife simulations yielded less than 13 clusters. The following clusters did not show in all jackknife simulations; Healthy home environment, Enabling environment, Tailored environment and Accessibility. Using full data and a cluster size of 11, these four clusters would be combined in *Physical environment* (*Healthy home environment* and *Enabling environment*) and *Accessibility* (*Tailored environment* and *Accessibility*).

## DISCUSSION

This study aimed to conceptualize healthy settings for people with ID. The combined experience of researchers involved in healthcare for people with ID and healthy settings researchers was capitalized to conceptualize healthy settings for people with ID. The study resulted in 13 clusters, which each make their own specific contribution to a health-promoting setting and encompass the physical environment, the social environment and societal preconditions. Several aspects—including a whole system approach (social, economic, policy and environmental), and values as equity and empowerment—of the settings approach (Dooris, 2009) are part of the concept map. In addition, the concept map highlights specific aspects of the settings in which people with ID live, work and engage.

Firstly, the five clusters related to the physical environment describe the physical resources of the setting, the interconnectivity between personal characteristics and place, and the home environment where the physical and the social environment merge. Physical resources that can support healthy living include resources indoors (*Healthy home environment)* and resources in the nearby area (*Enabling environment).* The contribution of these factors to individual lifestyles is supported by the literature. For example, accessibility of facilities for physical activity, esthetics, perceived nature or the local food environment are related to physical activity and dietary intake (Botchwey *et al.*, 2014; Keskinen *et al.*, 2018). Furthermore, the extent to which the environment is tailored (*Tailored environment*) and accessible (*Accessibility*) relates to universal design, including principles for designing the built environment in accordance with the needs of a wide variety of potential user groups (Steinfeld and Maisel, 2012). Specific needs of people with ID in relation to the built environment emanate from the high prevalence of mobility limitations (26%) and visibility limitations (19%) in this population (Nederlandse Vereniging van Artsen voor Verstandelijk Gehandicapten, 2012). If these needs are not taken into account, the built environment can increase the effect of having a disability on health (Eisenberg *et al.*, 2016). Other specific factors for people with ID include living with other persons with ID and having support staff around them, described in the cluster *Homely environment*. Feeling at home in one’s house relates to factors in the social environment.

Secondly, the five clusters related to the social environment describe the social network of people with ID and prerequisites for a health-promoting social network. The *Supportive network* includes family, friends, people in the community and care professionals. Care professionals are often involved in the lives of people with ID living in residential care facilities. People with ID often view these professionals as members of their social network (Kamstra, 2017). A health-promoting supportive network provides a sense of belonging and intimacy and helps people to be more competent and self-efficacious (Berkman, 1995). These prerequisites were mentioned in the clusters: *Values about healthy lifestyle*, *An open conversation*, *Confidence-building support* and *Encouraging support*. Statements within these clusters relate to enabling people by focusing on their strengths, adapting to their needs, including them in decision-making and providing them with personal space and independence. Empowering people to take active control of health determinants, one of the health promotion principles (WHO, 1986), is especially relevant to people with ID as their support and care has until a few decades ago been dominated by a protective atmosphere where rights to autonomy were often denied (Jenkinson, 1993).

Lastly, the clusters *Financial aspects*, *Healthcare and prevention* and *Opportunities to engage* relate to preconditions in society and are interconnected with both the physical and the social environment. The interaction and connections between components of a setting within components of other settings and the wider environment is reflected on in literature on the settings approach (Dooris, 2013; Bloch *et al.*, 2014). For people with ID specifically, the relationship between the physical environment and *Opportunities to engage* is underlined in McConkey’s study, which showed that the type of living accommodation of a person with ID has considerable influence on social inclusion (McConkey, 2007). Health-related policies (*Healthcare and prevention*) also are specifically important for people with ID, as such people have more health-related problems than the general population (Van Schrojenstein Lantman-de Valk and Walsh, 2009). Furthermore, *Financial aspects* are specifically important for people with ID on an individual level, as people with ID often have limited financial resources, and this is detrimental to their opportunities for healthy living, and for the governmental level including governmental budgets on specialized care for people with ID and social safety (Emerson, 2007).

Besides and beyond the 13 clusters, many statements provide guidance on how care providers for people with ID could facilitate health promotion. These include allocating funding for resources for health promotion, health promotion policies, access to health professionals, regular health checks and having employees who are educated about health promotion and know how to connect healthy lifestyles to daily routines. These factors align with the literature on organizational facilitators of healthy living for people with ID (Bergström *et al.*, 2014; Sundblom *et al.*, 2015; O’Leary *et al.*, 2018; unpublished results). As the core business of settings for people with ID is the provision of care, a culture change is needed whereby care providers for people with ID adopt a health promotion ethos (O’Leary *et al.*, 2018).

This study applied the settings approach as theoretical framework. Results of this study indicate different aspects of the whole systems approach including social, economic, policy and environmental factors. Furthermore, this study provides points for attention when applying the settings approach to settings in which people with ID engage. Firstly, this study highlights a health-promoting social network of people with ID as a prerequisite for change. What makes the social context of people with ID distinct is the limited ability of people with ID to address changes themselves and support needs from their social network. In practice this might be challenging as people with ID often have a small social network which they find difficult to maintain over the course of their life (Kamstra, 2017). Secondly, due to the heterogeneity of the population there is no ‘one size fits all’ regarding the physical context since there is a broad variety of adjustments needed. Lastly, connecting upward, meaning ensuring action on overarching determinants of health, is described as a way forward in the settings approach (Dooris, 2013). This strongly applies to people with ID since many people with ID face health inequities related to overarching determinants of health, of which income, social status and access to health services were mentioned in this study. This needs to be addressed on (inter)national level. In sum, this study provides challenges and directions for care providers, local and international policymakers to develop healthy settings for people with ID.

This study’s findings should be interpreted in light of a few limitations. The first relates to potential selection bias as a result of expert sampling. Although the sample size was relatively small, it is similar to that of other studies conducted using concept mapping (Rosas and Kane, 2012). Most clusters had low (*n* = 3) or moderate (*n* = 9) mean bridging values; the cluster *Opportunities to engage* had a high mean bridging value, which is a product of statements that were frequently grouped with items other than those within the cluster. Furthermore, this study reflects only perspectives of expert researchers and therefore lacks the perspective of people with ID, their guardians and caregivers.

A strength of this study is the additional sensitivity to sampling variation analysis, which we have not seen used before in similar studies. This analysis indicated 4 of the 13 clusters to be sensitive to sampling variation. An 11-cluster solution, where the 4 sensitive clusters are combined, would result in a cluster map that is less sensitive to sampling variation. We chose, however, to stay with the original 13-cluster map because of the stage of this research and our aim to develop a conceptual framework where a distinction between aspects of the physical environment and accessibility for healthy settings seem relevant. Future studies can investigate the empirical relevance of all clusters.

The multidisciplinary and international approach in which perceptions of researchers both in healthcare for people with ID and in healthy settings from 14 different countries are included is beneficial for the scope and applicability of the cluster map. These results can help to guide discussion with people with ID themselves about important factors for a healthy setting. In a future study, the views of people with ID will be gathered to complement the views of researchers, validate the results of this study and tailor the cluster map to more local applications.

## CONCLUSION

This study used concept mapping to conceptualize healthy settings for people with ID. The social environment, the physical environment and societal preconditions, and their interconnectivity with one another and with individuals in the setting, play an important role in healthy settings. Clusters not only reflect concepts already familiar in health promotion for the general population, but also indicate where tailoring is required for settings where people with ID live, work and engage. Factors specifically for healthy settings for people with ID include: (i) universal design of the physical environment, (ii) the role of care professionals in the social environment to empower people with ID, (iii) possibilities for care providers to contribute to a health-promoting setting and (iv) preconditions that allow people to engage in society. By identifying these factors, this study contributes to the limited knowledge on applying principles of healthy settings for people with ID. The identified factors that contribute to healthy settings for people with ID can be used put local and international policies on developing enabling environments for people with disabilities and decreasing health inequities in place.

## ETHICAL APPROVAL 

The study is conducted according to the principles of the Declaration of Helsinki (October 2013, 64th WMA General Assembly) and in accordance with the Dutch Personal Data Protection Act. Informed consent was obtained from all participants prior to data collection.

## Supplementary Material

daz054_Supplementary_Data
