# Supplementary material for: Towards healthy settings for people with intellectual disabilities
Source: Health Promot Int. 2019 Jun 26;35(4):661–70. doi: 10.1093/heapro/daz054 (PMC7414853; doi:10.1093/heapro/daz054)
Supplement: daz054_Supplementary_Data [file daz054_supplementary_data.zip › daz054-suppl_data/Appendix A.docx]

**Appendix A: Statements per cluster with accompanying bridging values (B) and mean importance (I)**

| **ID** | **Statement** | **B^1^** | **I^2^** |
| --- | --- | --- | --- |
| **Cluster 1: Healthy home environment** | | **0.07** | **3.79** |
| 34. | a place where I am sometimes afraid of people with whom I live | 0.20 | 3.48 |
| 50. | a comfortable setting with enough daylight, fresh air, and privacy | 0.05 | 4.06 |
| 63. | being attractively designed and having appropriate and enabling visual stimuli (nice views) | 0.01 | 3.53 |
| 69. | a kitchen to cook | 0.02 | 3.88 |
| 73. | a nice day center/workplace that I go to most days | 0.11 | 3.63 |
| 81. | a clean home (air, water, toilet, bathroom) | 0.00 | 4.47 |
| 88. | any other house in the neighborhood | 0.11 | 2.97 |
| 93. | living in a community that ensures accessible and universally designed parks, grocery stores, schools, and recreational activities | 0.08 | 4.25 |
| 95. | my own apartment (in shared accommodation) | 0.07 | 3.84 |
| 96. | a garden to grow food and flowers | 0.01 | 3.45 |
| 99. | a home that allows me to age in-place | 0.08 | 4.13 |
| **Cluster 2: Enabling environment** | | **0.14** | **4.17** |
| 15. | a place with lots to do/activities/challenges | 0.25 | 4.09 |
| 18. | nice areas nearby to walk or ride my bike | 0.19 | 4.00 |
| 26. | shared spaces to mingle in my community (neighborhood, school, shopping mall, supermarket, park, social clubs) | 0.16 | 4.31 |
| 65. | an environment that provides opportunities for active travel such as walking or cycling or using stairs (to day activities) | 0.07 | 4.28 |
| 83. | an accessible built environment and roads (safe crossing) | 0.05 | 4.16 |
| **Cluster 3: Homely environment** | | **0.21** | **3.82** |
| 11. | a home shared with other persons with intellectual disabilities and support staff | 0.28 | 2.84 |
| 19. | a nice place where I live with my family and friends | 0.23 | 4.22 |
| 28. | a sheltered setting | 0.14 | 2.97 |
| 58. | a place I can call home | 0.11 | 4.38 |
| 71. | a fun place to live and work, experience happiness | 0.27 | 4.28 |
| 80. | a place where I can bring friends | 0.24 | 4.23 |
| **Cluster 4: Tailored environment** | | **0.35** | **3.33** |
| 6. | taxi rides + waiting | 0.34 | 2.91 |
| 52. | no (public) transport nearby | 0.33 | 3.47 |
| 53. | somewhat isolated | 0.47 | 3.34 |
| 55. | having resources and an environment that has healthy food options and places to be physically active | 0.35 | 4.45 |
| 64. | internet/Facebook/smartphones/apps/technology | 0.42 | 3.41 |
| 87. | too noisy/crowded | 0.26 | 2.94 |
| 90. | a dull grey place/boring | 0.31 | 2.81 |
| **Cluster 5: Encouraging support** | | **0.36** | **4.05** |
| 24. | acceptance of my differences and able to adapt to my needs/resources | 0.35 | 4.19 |
| 31. | helping me exercise by exercising with me | 0.41 | 4.09 |
| 36. | having people who support me to make health lifestyle choices (discourage snacking and eat healthy food) | 0.25 | 4.13 |
| 38. | people who listen to me and take my complaints seriously | 0.21 | 4.44 |
| 40. | encourage me to do things rather than stop me doing things | 0.29 | 4.19 |
| 41. | a support group to help me get better health | 0.36 | 3.78 |
| 44. | (in the negative) avoiding expecting 'change' to be solely initiated by individuals themselves | 0.41 | 3.23 |
| 54. | communication flows/challenges (between staff) | 0.40 | 3.72 |
| 56. | my staff and family members advocate on my behalf with health professionals | 0.38 | 4.19 |
| 70. | having significant resource support or capacity (finance, skilled staff, supportive management) | 0.50 | 4.13 |
| 74. | respecting me and others with intellectual disabilities, and encouraging us to all respect one another | 0.38 | 4.50 |
| **Cluster 6: Supportive network** | | **0.41** | **4.12** |
| 25. | having family and friends who seek out health-related activities in which to participate | 0.37 | 4.25 |
| 29. | having people around me who notice if I am behaving differently or am sad, have pain or feel unwell | 0.36 | 4.13 |
| 62. | a lot of different caregivers, coaches, mentors, therapists, volunteers | 0.45 | 3.38 |
| 66. | sufficient support with daily living tasks (cooking, shopping, to get to places/appointments) | 0.44 | 4.13 |
| 67. | family, friends, and carers who promote health, physical activity, and social engagement | 0.31 | 4.44 |
| 68. | being in a relationship | 0.61 | 3.59 |
| 75. | I have people around me who are interested in who I am and I can trust and spend time with | 0.39 | 4.31 |
| 84. | having people (support, family, friends) near me who show role model behavior | 0.30 | 4.31 |
| 100. | surrounded by care providers who are educated and inspired to facilitate my environment, so that the healthy choice is the easy choice for me in every situation | 0.47 | 4.55 |
| **Cluster 7: Financial aspects** | | **0.45** | **3.86** |
| 2. | laws to protect me | 0.52 | 4.59 |
| 13. | cheap nutrition/diet | 0.54 | 3.63 |
| 32. | access to cheap healthy food | 0.36 | 3.88 |
| 35. | funding for adaptations/resources | 0.41 | 4.00 |
| 48. | having a job that has accessible and universally designed health and wellness activities for all employees | 0.44 | 4.00 |
| 78. | having alternatives to sugary drinks | 0.39 | 3.75 |
| 79. | too complex/not understandable/unpredictable/always changes | 0.51 | 3.16 |
| **Cluster 8: Confidence-building support** | | **0.49** | **4.11** |
| 14. | having personal space over how I live my life (make choices on my own health needs, food, sex) | 0.62 | 4.41 |
| 33. | user-friendly assistant devices (mobility, language) | 0.40 | 3.87 |
| 45. | having an opportunity to participate in religious activities that are inclusive of people with disabilities (e.g. physical access for people who are deaf or hard of hearing, blind or restricted vision, ramps) | 0.43 | 3.66 |
| 47. | a place to promote and enable my independence and support my life | 0.41 | 4.47 |
| 49. | cues in daily activity and food (nudging) e.g. taking stairs as a cue for doing exercise training | 0.52 | 3.91 |
| 59. | being free from exposure to discrimination and violence | 0.37 | 4.78 |
| 72. | routine/patterned/predictable/comprehensible | 0.46 | 3.44 |
| 82. | people in the community (neighbors, customer, hairdresser, beautician, staff in supermarket, postman, strangers, celebrities) | 0.62 | 4.09 |
| 85. | feeling safe, comfortable, supported, and engaged with my non-disabled friends | 0.60 | 4.41 |
| 92. | the leaders/management of my shared accommodation have employed care providers designated to be responsible for physical activity and some for nutrition | 0.49 | 4.09 |
| **Cluster 9: An open conversation** | | **0.50** | **4.14** |
| 27. | talk to me about issues (death and dying, consequences of poor health, having a disability) | 0.51 | 3.91 |
| 39. | my care providers have guidelines on how to provide health promotion in our setting | 0.69 | 4.22 |
| 43. | having conversations about health in an informal way and incorporating our ideas | 0.54 | 3.88 |
| 46. | increasing my self-efficacy with manageable but demanding tasks | 0.45 | 4.22 |
| 51. | focus on my talent and strengths and abilities, not only my deficits | 0.39 | 4.47 |
| 91. | being able to ask questions | 0.45 | 4.13 |
| **Cluster 10: Values about healthy lifestyle** | | **0.56** | **4.08** |
| 5. | enabling me to make informed choices about my own health | 0.57 | 4.63 |
| 8. | being spoken to/not at and included in decision making | 0.52 | 4.50 |
| 9. | connecting healthy eating and exercise to daily routines in a fun way | 0.56 | 4.19 |
| 16. | having a clear role/purpose/value | 0.53 | 4.13 |
| 17. | education about ill health and healthy living (eating, cooking, exercise, personal hygiene) | 0.59 | 4.13 |
| 30. | it is ok for me to be very overweight | 0.6 | 2.72 |
| 89. | emphasizing general well-being as a goal | 0.57 | 4.26 |
| **Cluster 11: Healthcare and prevention** | | **0.65** | **4.00** |
| 4. | having regular access to health professionals (doctors, nurses, specialists) | 0.62 | 4.53 |
| 10. | ensuring guidelines relating to problems with eating, drinking, and swallowing are followed | 0.67 | 3.88 |
| 21. | medical places (GP's office, dentist, hospital) | 0.75 | 4.03 |
| 22. | (in the negative) seeing 'health' in traditional topic and behavioral terms (diet, activity, etc.) | 0.64 | 3.13 |
| 57. | medical people: nurses, doctors, pediatricians, dentist, dietician | 0.57 | 3.88 |
| 60. | prevention (sunscreen, alcohol, stress, sleep) | 0.62 | 3.94 |
| 61. | diagnosis of real medical issues and preventing unnecessary medication | 0.60 | 4.28 |
| 76. | that politicians always think of us when they are planning new concepts/policies in the municipality | 0.76 | 4.29 |
| 77. | ensuring that I have an annual health check | 0.64 | 4.06 |
| **Cluster 12: Accessibility** | | **0.65** | **3.78** |
| 3. | not allowed/safe to go out on my own | 0.75 | 3.87 |
| 7. | sport activities (dancing, zumba, etc.) | 0.76 | 3.88 |
| 12. | a place to learn | 0.57 | 3.78 |
| 20. | events/parties/festival with healthy food and drinks | 0.65 | 3.53 |
| 42. | obstacles (physical + abstract) | 0.63 | 3.39 |
| 86. | do a lot of meaningful activities and participate | 0.55 | 4.26 |
| **Cluster 13: Opportunities to engage** | | **0.91** | **3.66** |
| 1. | allowing me the opportunity to make choices regarding where I live and with whom I live | 1.00 | 4.44 |
| 23. | not as rich as other people | 0.99 | 2.75 |
| 37. | not making my own money/dependence on benefits (social security payments, food stamps) | 0.73 | 3.16 |
| 94. | having less say how things are run | 0.90 | 3.38 |
| 97. | participate in mainstream programs | 0.83 | 4.06 |
| 98. | (not) giving me the opportunities to learn, work, and have social relationships | 1.00 | 4.16 |

^1^ B=bridging value between 0 and 1; ^2^ I=importance (rated on a 5-point Likert scale)
