# Supplementary figures and images for: Towards healthy settings for people with intellectual disabilities
Source: Health Promot Int. 2019 Jun 26;35(4):661–70. doi: 10.1093/heapro/daz054 (PMC7414853; doi:10.1093/heapro/daz054)

**Appendix B: Point map visualizing the relationship and proximity of statements to one another**

**
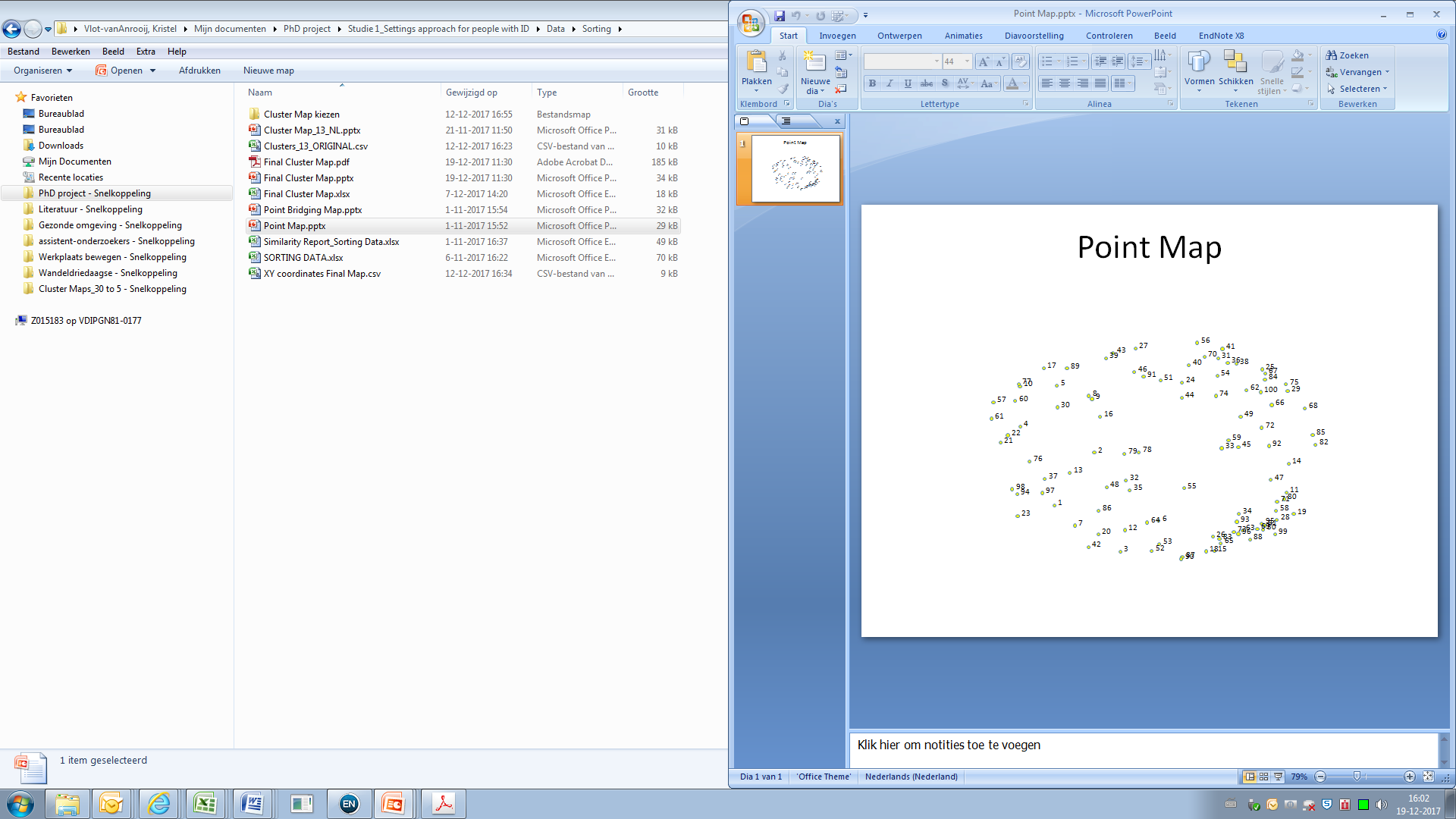
**

Supplement: daz054_Supplementary_Data [file daz054_supplementary_data.zip › daz054-suppl_data/Appendix B.docx]
